# Salt Replacement Changed the Bacterial Community Composition and Physicochemical Characteristics of Sodium-Reduced Fermented Sausages during Fermentation and Ripening

**DOI:** 10.3390/foods10030630

**Published:** 2021-03-17

**Authors:** Ying Zhang, Yifan Zhang, Xing Zhou, Shibo Wang, Pinglan Li

**Affiliations:** Key Laboratory of Precision Nutrition and Food Quality, College of Food Science and Nutritional Engineering, China Agricultural University, Beijing 100083, China; zy920506@cau.edu.cn (Y.Z.); zhangyifan183@163.com (Y.Z.); KingChou@cau.edu.cn (X.Z.); wangshibo@cau.edu.cn (S.W.)

**Keywords:** salt replacement, potassium chloride, calcium ascorbate, calcium glutamate, fermented sausages

## Abstract

The impact on fermented sausages with 25% replacement of the sodium chloride content by 14% potassium chloride, 10% calcium ascorbate and 1% calcium glutamate during fermentation and ripening was evaluated based on the bacterial community composition and physicochemical and sensory characteristic analysis. Our results showed that the use of salt replacement varied the composition of the bacterial community and reduced the diversity of that in sodium-reduced fermented sausages. Moreover, the decrease in pH and the moisture content of fermented sausages with salt replacement accelerated the drying and ripening processes. The texture profile and color analysis did not reveal marked differences between normal fermented sausages and sodium-reduced products with salt replacement; however, salt replacement reduced resilience and lightness of fermented sausages. In addition, as shown in the principal component analysis, the comprehensive parameters of the fermented sausages with salt replacement were similar to those of normal salt products. These results indicate that the complex blends of salt replacement have great potential to be used to produce sodium-reduced fermented sausages.

## 1. Introduction

Excessive salt (sodium chloride, NaCl) intake is associated with an increased risk of high blood pressure (HBP, or hypertension), cardiovascular disease and some types of cancer [1,2] and is widely considered by the public. Therefore, over the past decades, the World Health Organization (WHO) and the U.S. Food and Drug Administration (FDA) have recommended reducing salt consumption by establishing programs [3,4]. Because of the current sodium-reduction awareness of politicians and consumers, it has become imperative to reduce the sodium content in processed food, especially in processed meat products. Of all processed meat products, dry fermented sausages are among those with the highest sodium content due to excessive water loss during maturation [5]. However, salt reduction of dry fermented sausages is a huge challenge because salt provides many important functions that contribute to color, texture, flavor and shelf life safety of dry fermented sausages [6,7].

Consequently, much research has been conducted to identify ingredients to replace the functionality of NaCl in meat products, especially focusing on the use of other chloride salts. Potassium chloride (KCl, E508) provides one of the most direct salt replacements due to the similarity in the molecular composition and is commonly used in processed meat products without affecting their texture, color and microbiological characteristics [8,9,10,11,12]. Bidlas and Lambert [13] reported that KCl has an equivalent antimicrobial effect as NaCl against some pathogens. Moreover, potassium intake reduces the risk of hypertension and cardiovascular diseases [14,15]. However, negative sensory attributes such as bitter, metallic and astringent tastes have been reported when KCl is used as the sole salt substitute [11,16,17]. Therefore, searching for ingredients that can reduce or prevent adverse sensory effects by using KCl is of vital importance in developing low-salt processed meat products.

Calcium ascorbate (Vc-Ca, E302) is a possible substitute for salt and is employed as an antioxidant in processed milk and meat products, which also accelerates the curing reaction and develops a stable cured color in cured meat products [18,19,20]. Moreover, calcium glutamate (E623) has a salty and umami taste as a flavor enhancer, which enhances the acceptability of low-salt sausages [21]. In addition, Vc-Ca and calcium glutamate as salt replacements increase the calcium intake of consumers of meat products. As reported previously, calcium increases the hardness of fermented sausages [22]. However, few studies have evaluated the complex blends of salt replacement with such ingredients in fermented sausages.

Hence, in this study, we aim to find out the potential effect of 25% replacement of the NaCl content by 14% KCl, 10% Vc-Ca and 1% calcium glutamate on the bacterial community composition and physicochemical and sensory characteristics of the fermented sausages during fermenting and ripening. This study provides the basis for potential applications of complex blends of salt replacement to be used for low-salt fermented sausage production.

## 2. Materials and Methods

### 2.1. Preparation of the Fermented Sausages

The fermented sausages were prepared according to Zhang et al. [23] with some modifications of the ingredients. All meat and ingredients were purchased from Wumart supermarket (Haidian district, Beijing, China). The ingredients in the traditional formulation were added to the mixture of 70% chopped lean pork meat and 30% pork back fat mass as follows: powdered milk 10 g/kg, glucose 3.5 g/kg, cracked black pepper 1 g/kg, garlic 0.15 g/kg, cinnamon 0.1 g/kg, ascorbate 0.5 g/kg, sodium nitrite 0.1 g/kg and white wine 3 mL/kg. In the normal fermented sausages (N group), 30 g/kg salt (NaCl) was added to the mixture. The low-salt fermented sausages (L group) had a 25% reduction in salt (22.5 g/kg was added). In the partial replacement of salt in fermented sausages (R group), salt (22.5 g/kg) and the optimized formula (data shown in Figure S1), i.e., KCl 4.2 g/kg, Vc-Ca 3 g/kg and calcium glutamate 0.3 g/kg, were added. All fermented sausages were inoculated with a recommended commercial starter culture composed of *Pediococcus pentosaceus* and *Staphylococcus xylosus* (F-1 BactofermTM, Chr-Hansen, Denmark) for traditionally fermented meat products. The stuffed sausages with a diameter of 45 mm were hung in a temperature-controlled room for 36 days. Fermentation was performed at 25 °C and 94% relative humidity (RH) for the first 4 days. For the ripening process, the temperature was set at 15 °C, 94% RH for 14 days and 80% RH for 14 days. Samples were taken on days 0, 4 (end of fermentation), 12, 20, 28 and 36 (end of ripening).

### 2.2. Chemical Analysis

The pH was determined by homogenizing 5 g of minced fermented sausages in 50 mL of distilled water and using a pH meter (MP230, Mettler Toledo, Switzerland). The weight loss was calculated as a percentage of the reduced weight to the weight of fermented sausages on day 0. The moisture content was measured according to the high-temperature drying method by oven at 105 °C. The water activity (a_w_) was determined using a HygroLab-C1 (Rotronic, NY, NY, USA). The chloride content of the fermented sausages was confirmed by the Volhard method as shown in ISO 1841-1:1996 [24]. The 10 g minced sample with 100 mL hot water was boiled for 15 min. The Carrez reagent was added to precipitate proteins for 30 min. The 20 mL filtrate was added to 5 mL dilute nitric acid, 1 mL ammonium iron(Ⅲ) sulfate solution as indicator and 20 mL silver nitrate solution. Excess silver nitrate was titrated with standard potassium thiocyanate solution. The sodium content was measured by ion-selective electrode method according to AOAC Official Method 976.25:1995 [25]. Briefly, the mV potential of a 10 g minced sample with 100 mL buffer was determined by ion meter (PXSJ-227L, INESA, Shanghai, China). The nitrite content was determined by photometric measurement according to ISO 2918:1975 [26].

### 2.3. Texture Profile Analysis (TPA) and Color Measurement

Texture profile analysis (TPA) was conducted using a texture analyzer by TAXT software (TA-XT plus; Stable Micro Systems, Godalming, UK) fitted with a cylindrical probe (P/50, 50 mm stainless). Sausages were cut into cylindrical shapes (height 1 cm, diameter 30 mm) to fit on the platform. The double compression cycle test was conducted up to 50% of the height of the sample. TPA parameters were set at a pre-test speed of 2 mm/s, test speed of 1 mm/s post-test speed of 2 mm/s, probe return speed of 5 mm/s and contact force of 5 g. The determined parameters were hardness, adhesiveness, cohesiveness, springiness, chewiness and resilience. Color measurement of the fermented sausages was determined using a colorimeter (CR-40, Minolta Camera, Japan). The values of L* (lightness), a* (redness) and b* (yellowness) were recorded from three different areas of the samples.

### 2.4. Microbiological Analysis

Microbiological analysis was according to the method described by Ravyts et al. [27]. Briefly, 10 g of the fermented sausages was aseptically transferred into a sterile plastic bag and homogenized with 90 mL of sterilized saline by a stomacher (Stomacher 400; Seward, Worthington, UK) for 10 min. The counts of total viable bacteria, lactic acid bacteria (LAB), *Staphylococcus* and *Micrococcus* and Enterobacteriaceae were determined by plate count agar, de Man, Rogosa, and Sharpe (MRS) agar, mannitol salt agar (MSA) and violet red bile dextrose agar (VRBDA) at 37 °C for 48 h, respectively. The results were expressed as Log CFU/g.

### 2.5. Bacterial DNA Extraction, Polymerase Chain Reaction (PCR) and Sequencing of the 16S rDNA

Bacteria in samples were extracted according to Xiao et al. [28] and bacterial DNA was extracted with a GenElute™ Kit (Tiangen Biotech, Beijing, China). 

The universal primers 338F and 806R were used to amplify the V3-V4 regions of bacterial 16S rDNA genes from 30 samples (10 samples × 3 replicates) [29], and a thermal cycling protocol was performed as described by Zhang et al. [23]. The PCR amplicons were purified and quantified using the AxyPrep DNA Gel Extraction Kit (Axygen, Union, CA, USA) and QuantiFluor™-ST (Promega, Madison, WI, USA), respectively. A high-quality library for high-throughput pyrosequencing was constructed using the TruSeq^®^ DNA Library Prep Kit (Illumina, San Diego, CA, USA). The purified PCR amplicons with the generation of 2 × 300 base pairs (PE300) were sequenced on the Illumina MiSeq platform by Majorbio Bio-Pharm (Shanghai, China).

### 2.6. OTU-Based Bacterial Diversity Analysis

The raw reads obtained were processed using QIIME 1.17 [30]. The quality-filtered sequences were clustered into operational taxonomic units (OTUs) with a 97% similarity threshold using Usearch 7.0. The OTUs were classified with a 70% confidence threshold using RDP Classifier [31] against the Silva reference 16S rRNA gene database. The alpha diversity (the observed OTUs, ACE, Chao1, Simpson, Shannon and Goods coverage indexes) was determined using Mothur v.1.30.1 with a 97% similarity. The visualized principal coordinates analysis (PCoA) of beta diversity was calculated by weighted and unweighted UniFrac distances.

### 2.7. Statistical Analysis

All samples were carried out in triplicate (three independent productions). The statistical significance (*p* < 0.05) was analyzed by one-way ANOVA and Tukey’s post hoc test. Principal component analysis (PCA) was performed to visualize differences in multiple parameters of samples among all groups. The first two principal components were used for calculating the PCA models. SPSS 20 was used for all statistical analyses.

## 3. Results and Discussion

### 3.1. Chemical Analysis

**Figure 1:**
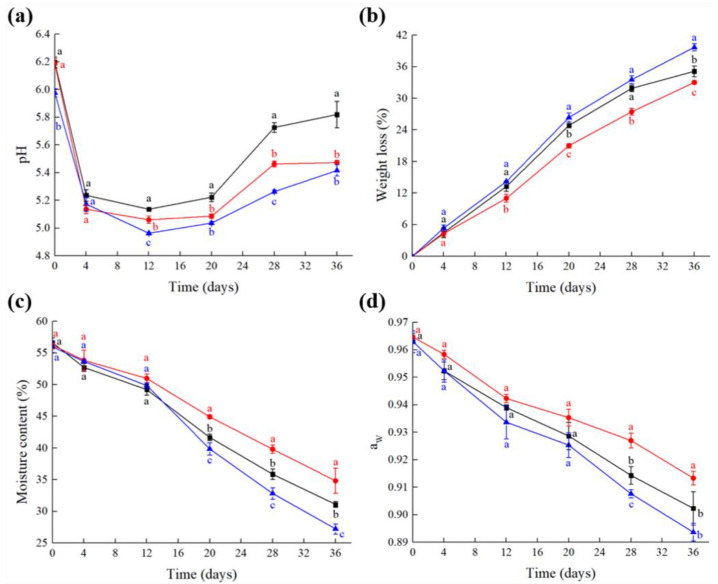
The pH (**a**), weight loss (**b**), moisture content (**c**) and a_w_ (**d**) in the fermented sausages. (■) N: fermented sausages with 3% NaCl (reference), (●) L: fermented sausages with 2.25% NaCl (25% reduction), (▲) R: fermented sausages with partial replacement of 25% NaCl. The different lowercase letters indicate significant difference (*p* < 0.05) between the samples by Tukey’s test.

The pH, weight loss, moisture content and a_w_ of the fermented sausages during fermentation and ripening were determined (Figure 1). As shown in Figure 1, the pH of the fermented sausages decreased first and then increased in every group during fermentation and ripening. Other published studies showed that in fermented sausages during ripening, the increase in pH is related to the decrease in electrolyte dissociation and/or an increase in the concentration of buffering proteins and the formation of ammonia [32,33]. The pH in the R group was obviously lower than that in the N and L groups during the processing of the fermented sausages, which was mainly related to the addition of Vc-Ca and calcium glutamate. As some previous studies reported, the presence of calcium reduced the pH of dry fermented sausages [19,34], while Gelabert et al. [35] reported that using KCl as a NaCl replacement did not severely change the pH of the fermented sausages. In this work, the lower pH of the fermented sausages with salt replacement was beneficial for inhibiting the growth of spoilage bacteria and enhancing the drying speed [7]. Moreover, due to moisture evaporation, the moisture content and a_w_ decreased, and weight loss increased in all groups during fermentation and ripening. On day 36, the weight loss, moisture content and a_w_ in the R group with the faster drying speed were significantly different from those in the other two groups (*p* < 0.05). The results showed that salt replacement accelerated the drying and ripening processes of the fermented sausages.

During fermentation and ripening of the fermented sausages, the chloride, sodium and nitrite contents were measured (Table 1). The chloride and sodium contents in all treatments increased significantly with gradual water loss (*p* < 0.05). The chloride content in the R group was significantly higher than that in the L group (*p* < 0.05), which showed a proportional increase with the addition of KCl. In comparison with mature normal fermented sausages (N group), the sodium content in f the R group was only 1505.22 ± 11.10%, which was reduced by approximately 25%. These low-salt fermented sausages provide healthier characteristics for reducing sodium intake. In addition, the nitrite content in all groups decreased significantly (*p* < 0.05) during fermentation and ripening. The results reflected that the nitrite degradation rate in the fermented sausages with salt replacement was significantly faster than that in the other two treatments (*p* < 0.05), which was mainly related to ascorbate addition [6,36].

### 3.2. TPA and Color Analysis

The instrumental texture analysis profile and color analysis of the fermented sausages were shown in Table 2. During fermentation and ripening of the fermented sausages, hardness, cohesiveness and chewiness increased and adhesiveness decreased significantly (*p* < 0.05), which were related to water loss and drying. As reported previously, the replacement of NaCl may change the texture of the fermented sausages [37,38,39], which contributes to the solubilization and diffusion of muscle myofibrillar proteins by NaCl [40]. In this work, no difference in adhesiveness, cohesiveness or springiness was found among all groups at the end of ripening (*p* > 0.05). However, the hardness and chewiness in the final products of the R group showed a significant increase compared with those of the low-salt control (*p* < 0.05), and there was no significant difference between the R and N groups on day 36 (*p* > 0.05). In addition, treatment with salt replacement significantly reduced the resilience of the final fermented sausages compared with the normal salt control (*p* < 0.05).

Color is an important parameter of the sensory properties that can be affected by NaCl replacement in processed meat [12,19,41]. The results in Table 2 show that the final fermented sausage products with salt replacement had significantly reduced lightness (L*, *p* < 0.05) and slightly increased redness (a*) and yellowness (b*).

### 3.3. Microbiological Analysis

**Figure 2:**
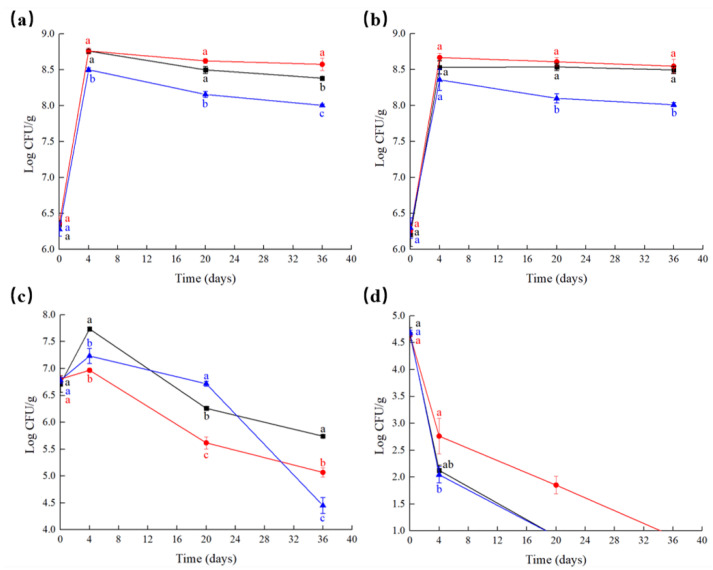
Evolution microflora of the fermented sausages. (**a**): Total viable bacterial counts, (**b**): lactic acid bacteria (LAB) counts, (**c**): *Staphylococcus* and *Micrococcus* counts, (**d**): Enterobacteriaceae counts. (■) N: fermented sausages with 3% NaCl (reference), (●) L: fermented sausages with 2.25% NaCl (25% reduction), (▲) R: fermented sausages with partial replacement of 25% NaCl. The different lowercase letters indicate significant difference (*p* < 0.05) between the samples by Tukey’s test.

The microbial counts of the fermented sausages during fermentation and ripening are presented in Figure 2. The initial counts of total viable bacteria, LAB, *Staphylococcus* and *Micrococcus* and Enterobacteriaceae in fermented sausages were approximately 6.30, 6.25, 6.75 and 4.65 Log CFU/g, respectively. As shown in Figure 2a,b, the total viable bacterial and LAB counts in all groups increased during fermentation and slightly dropped in the range of 8.00 to 8.76 Log CFU/g during ripening. However, salt replacement significantly reduced the total viable bacterial and LAB counts in the final fermented sausages compared with the normal and salt-reduced samples (*p* < 0.05). Moreover, the maximum *Staphylococcus* and *Micrococcus* counts were 7.74 ± 0.04 Log CFU/g in the N group after fermentation, as shown in Figure 2c. However, the *Staphylococcus* and *Micrococcus* counts in the R group were significantly lower than those in the N and L groups on day 36 (*p* < 0.05). In addition, significant decreases in Enterobacteriaceae counts in three groups during 36 days of processing (*p* < 0.05) are shown in Figure 2d. These results suggest that salt replacement has an antimicrobial effect on pathogenic bacteria, which also reduces starter counts, especially *Staphylococcus* and *Micrococcus* counts. As previously stated, KCl had a similar evolution of LAB and aerobic mesophile counts as NaCl and increased the growth of Micrococcaceae in fermented sausages [35]. We inferred that the growth of *Staphylococcus* and *Micrococcus* can be inhibited by Vc-Ca and calcium glutamate in fermented sausages, and we are working on related research.

### 3.4. Bacterial Richness and Diversity Analysis

In total, 1,523,684 high-quality and valid sequences were collected across all 30 samples of the fermented sausages, with an average sequence length of 428-bp. Table 3 displays the alpha diversity of the bacterial community structure of samples involving the observed OTUs, ACE, Chao1, Simpson, Shannon and Goods coverage indexes. The Goods coverage index in all samples exceeded 0.99, indicating that almost all bacteria in the samples were detected. During fermentation and ripening, the decrease in the observed OTUs, ACE, Chao1 and Shannon indexes and the increase in the Simpson index indicated a decrease in bacterial community richness and diversity in fermented sausages processing. Moreover, there was no significant difference among the three groups in the observed OTUs, ACE and Chao1 indexes at the end of ripening (*p* > 0.05), which suggests that salt replacement had no effect on bacterial community richness in the fermented sausages. In addition, the Simpson index in the R group was significantly higher and the Shannon index in the R group was significantly lower than that in the normal salt control on day 36 (*p* < 0.05). The results showed a decrease in the diversity of bacterial community in the sausages fermented with salt replacement.

### 3.5. Bacterial Community Composition

**Figure 3:**
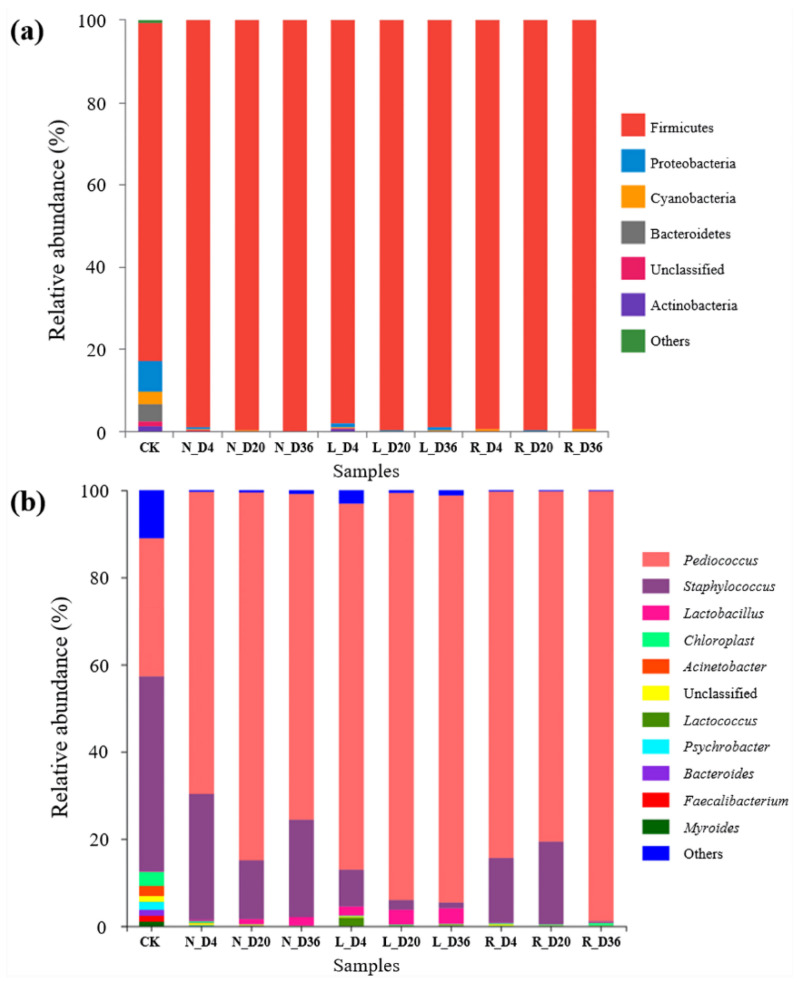
Relative abundance of the bacterial community based on 16S rDNA sequencing at the phylum (**a**) and genus (**b**) levels in fermented sausages. CK: initial fermented sausages (unfermented), N: fermented sausages with 3% NaCl (reference), L: fermented sausages with 2.25% NaCl (25% reduction), R: fermented sausages with partial replacement of 25% NaCl. The marks “D4, D20 and D36” represent the processing times (days).

The relative abundance of the bacterial community of the fermented sausages at the phyla level was shown in Figure 3a. In the initial fermented sausages, 96.87% of bacteria belonged to the phyla Firmicutes (82.27 ± 3.23%), Proteobacteria (7.44 ± 0.49%), Bacteroidetes (4.05 ± 2.05%) and Cyanobacteria (3.12± 0.91%). Furthermore, Firmicutes was the most abundant phylum, and the relative abundance of Firmicutes exceeded 97% in all groups after fermentation, indicating that the starter cultures of LAB and *Staphylococcus* had a dominant position in fermented sausages. However, after fermentation, all treatments in the composition of bacterial community at the phylum level have no obvious difference.

The composition analysis of the bacterial community in the fermented sausages at the genus level is shown in Figure 3b. In the initial fermented sausages, the genera *Pediococcus*, *Staphylococcus*, *Lactobacillus*, *Acinetobacter*, *Psychrobacter* and *Myroides* were the most representative bacteria. Moreover, LAB and *Staphylococcus* within Firmicutes were abundant genera, accounting for more than 90% in sausages after fermentation, which has been reported previously [23,42]. Compared with the N and L groups, *Pediococcus* composed the vast majority of the bacterial community of the fermented sausages in the R group at the end of processing, which illustrated that salt replacement controlled the growth of almost all bacteria but only slightly inhibited that of *Pediococcus*. As shown by the above results of microbiological plate colony counting, we inferred that Vc-Ca and calcium glutamate have antibacterial characteristics.

To understand the diversity of the bacterial community among all samples, a heatmap of the bacterial community structure and composition of the top 25 genera was shown in Figure S2a, which revealed the differences in the top genera between initial sausages and other fermented sausages at different processing stages. In addition, the relative abundances of the top significant genera in the samples are shown in Figure S2. According to Figure S2a,b, the relative abundance of *Pediococcus* in all groups increased significantly after fermentation and remained relatively stable until the end of ripening (*p* < 0.05), while *Staphylococcus* showed a slow decrease during fermentation and ripening. In fermented sausages, *Lactobacillus* and *Weissella* have been considered the principal LAB [23,43]. As detailed in Figure S2c,d, the relative abundance of *Lactobacillus* in the L group was higher than that in the other two groups, while the relative abundance of *Weissella* in the N group was higher after fermentation. These results illustrated that salt replacement inhibited the growth of almost all LAB in the fermented sausages.

### 3.6. Comparative Analysis of Bacterial Community

**Figure 4:**
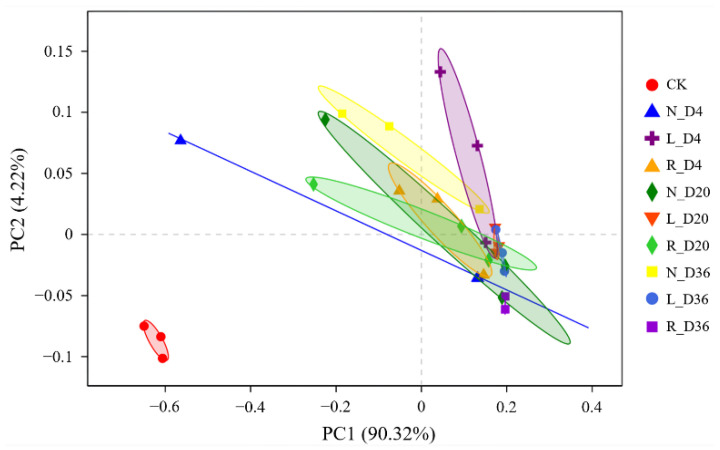
PCoA based on operational taxonomic units (OTUs) of the bacterial community in samples of the fermented sausages. CK: initial fermented sausages (unfermented), N: fermented sausages with 3% NaCl (reference), L: fermented sausages with 2.25% NaCl (25% reduction), R: fermented sausages with partial replacement of 25% NaCl. The marks D4, D20 and D36 represent the fermentation and ripening times (days).

The beta diversity among 30 samples (10 groups × 3 replicates) was analyzed and clustered by PCoA (Figure 4). The results showed that all samples were divided into two segments, and one segment consisting of initial sausage samples was separated from other samples of another segment. As reported previously, there was a considerable difference in the bacterial community structures between unfermented and fermented sausages [22]. To better investigate the difference in the bacterial community in the sausages of the three groups after fermentation, specific PCoA was performed (Figure S3). From Figure S3a–c, the fermented sausage samples of the three groups were distributed on days 4, 20 and 36, indicating that salt reduction or replacement had an effect on the bacterial community of the fermented sausages.

**Figure 5:**
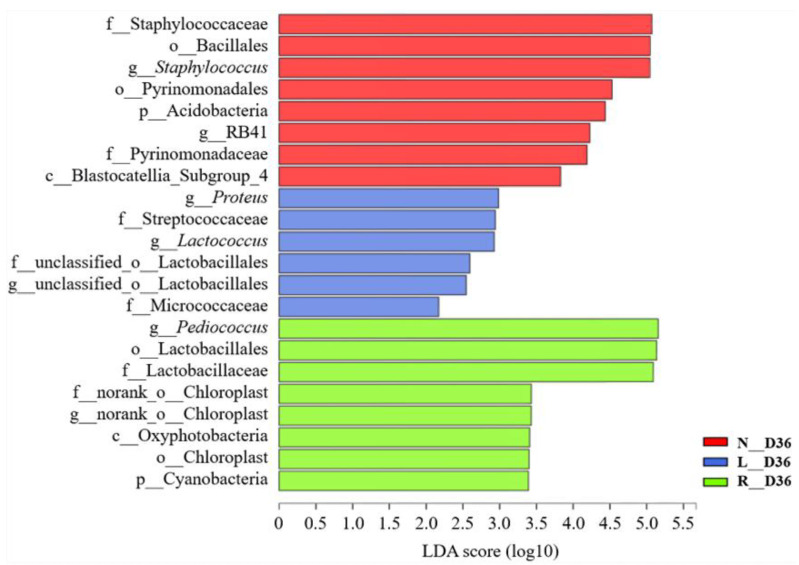
Horizontal bar chart of bacterial markers among the three groups (N, L and R) of samples on day 36 based on linear discriminant analysis (LDA). Bacterial taxa were significantly discriminant taxa from the phylum to genus levels with LDA scores higher than 2.0. The letters o_, f_ and g_ represent bacterial order, family and genus, respectively. N: fermented sausages with 3% NaCl (reference), L: fermented sausages with 2.25% NaCl (25% reduction), R: fermented sausages with partial replacement of 25% NaCl.

Bacterial markers among the three groups of the final fermented sausage samples were evaluated and analyzed by LDA (Figure 5). A total of 22 bacterial clades that were significantly discrepant in relative abundance among the three treatments were identified. As a result, Bacillales, Pyrinomonadales, Pyrinomonadaceae, Staphylococcaceae and *Staphylococcus* in the N group; Micrococcaceae, Streptococcaceae, *Lactococcus* and Proteus in the L group; and Lactobacillales, Lactobacillaceae and *Pediococcus* in the R group were the potential biomarkers in relative abundance for distinguishing among all groups.

### 3.7. Principal Component Analysis (PCA)

**Figure 6:**
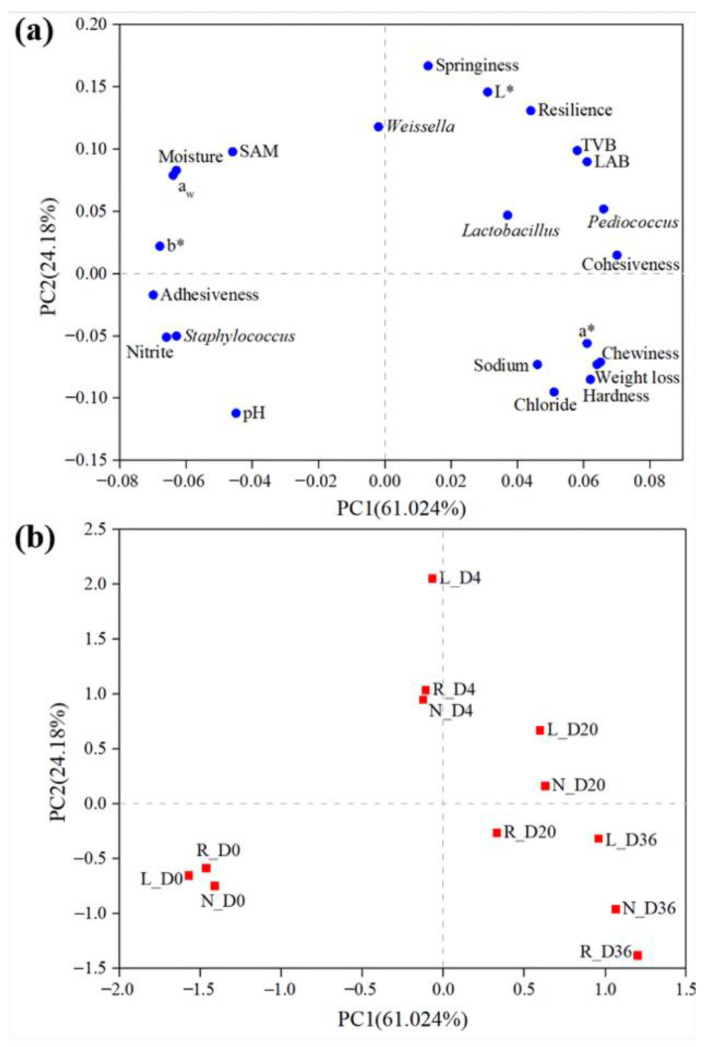
PCA plots of multiple parameters (**a**) and samples of the fermented sausages (**b**). The ● represents multiple parameters. TVB: total viable bacterial counts, SAM: *Staphylococcus* and *Micrococcus* counts. The ■ represents the samples. N: fermented sausages with 3% NaCl (reference), L: fermented sausages with 2.25% NaCl (25% reduction), R: fermented sausages with partial replacement of 25% NaCl. The marks D0, D4, D20 and D36 represent the fermentation and ripening times (days).

PCA was performed to analyze the linear correlation of multiple parameters involving pH, weight loss, a_w_, moisture, chloride, sodium and nitrite contents, TPA (hardness, adhesiveness, cohesiveness, springiness, chewiness and resilience), color analysis (L*, a* and b*) and microbiological analysis (plate colony counting and relative abundance of genera) in fermented sausages during processing (Figure 6). The results of the PCA indicated that the first and second principal components (PC1 and PC2) explained 85.204% of the total variability (61.024% PC1 and 24.18% PC2) contained in the original variables. As shown in Figure 6a, all characteristics of the fermented sausages were located within the plane of two principal components. In terms of PC1 (the primary axis), cohesiveness and the relative abundance of *Lactobacillus* and *Pediococcus* were on the positive side of PC1, while adhesiveness and b* were located on the negative axis of the first principal component. According to the cluster of multiple parameters, the moisture content and a_w_ were correlated with the *Staphylococcus* and *Micrococcus* counts. Moreover, the nitrite content of the fermented sausages showed a correlation with the relative abundance of *Staphylococcus*. In addition, chewiness, hardness and a* were clustered with weight loss and sodium and chloride contents. Figure 6b showed the different groups of sausages during fermentation and ripening distributed in two-dimensional space with PC1 and PC2. The initial sausages (N_D0, L_D0 and R_D0) were away from other samples and clustered together. Moreover, the final fermented sausages with salt replacement (R_D36) was closest to the normal salt control, indicating that the application of complex blends of salt replacement in this study was effective in the fermented sausages.

## 4. Conclusions

In conclusion, our work showed that the use of complex blends of salt replacement containing KCl, Vc-Ca and calcium glutamate was a reliable and effective strategy of sodium reduction in fermented sausages. The use of salt replacement altered the composition of bacterial community and reduced the diversity of that in sodium-reduced fermented sausages. Moreover, the decrease in the pH and moisture content of the sausages fermented with salt replacement accelerated drying and ripening processes. The texture profile and color analysis did not reveal marked differences between normal fermented sausages and reduced-sodium products with salt replacement; however, salt replacement reduced resilience and lightness (L*). In addition, as shown by the PCA, the comprehensive parameters of the fermented sausages with salt replacement were similar to those of normal salt products. Therefore, complex blends of salt replacement containing KCl, Vc-Ca and calcium glutamate have great potential to be applied to produce sodium-reduced fermented sausages. Future research on the correlations between microbial metabolites and volatile flavor in fermented sausages with salt replacement during fermentation and ripening should be taken.

## Figures and Tables

**Table 1:** Evaluation of chloride, sodium and nitrite contents of the fermented sausages.

	Time (Days)	Samples		
		N	L	R
Chloride (%)	0	1.87 ± 0.03 ^a^	1.38 ± 0.07 ^c^	1.65 ± 0.04 ^b^
	4	2.00 ± 0.06 ^a^	1.44 ± 0.06 ^c^	1.73 ± 0.05 ^b^
	20	2.37 ± 0.17 ^a^	1.72 ± 0.08 ^b^	2.28 ± 0.13 ^a^
	36	2.79 ± 0.14 ^a^	2.05 ± 0.10 ^b^	2.73 ± 0.10^a^
Sodium (mg/100 g)	0	1286.96 ± 13.11 ^a^	891.25 ± 7.28 ^b^	901.76 ± 3.83 ^b^
	4	1351.22 ± 21.63 ^a^	934.69 ± 10.56 ^b^	959.98 ± 15.43 ^b^
	20	1708.84 ± 21.58 ^a^	1119.44 ± 23.23 ^c^	1237.69 ± 23.37 ^b^
	36	1998.77 ± 27.50 ^a^	1326.65 ± 29.35 ^c^	1505.22 ± 11.10 ^b^
Nitrite (mg/kg)	0	99.36 ± 1.00 ^a^	99.09 ± 1.35 ^a^	99.80 ± 1.34 ^a^
	4	14.53 ± 0.61 ^b^	27.81 ± 0.39 ^a^	10.50 ± 0.50 ^c^
	20	8.55 ± 0.33 ^b^	10.59 ± 0.92 ^a^	7.61 ± 0.25^b^
	36	6.62 ± 0.39 ^b^	7.53 ± 0.23 ^a^	6.77 ± 0.34 ^ab^

N: fermented sausages with 3% NaCl (reference), L: fermented sausages with 2.25% NaCl (25% reduction), R: fermented sausages with partial replacement of 25% NaCl. The average ± standard deviation followed by different lowercase letters indicate significant difference (*p* < 0.05) between the samples by Tukey’s test.

**Table 2:** Evaluation of TPA and color test of the fermented sausage.

	Time (Days)	Samples		
		N	L	R
Hardness (N)	0	2.65 ± 0.07 ^a^	2.20 ± 0.51 ^a^	2.85 ± 0.63 ^a^
	4	9.13 ± 0.90 ^a^	8.51 ± 1.14 ^a^	9.39 ± 1.04 ^a^
	20	39.03 ± 2.44 ^a^	38.25 ± 2.24 ^a^	40.99 ± 5.52 ^a^
	36	79.56 ± 1.98 ^a^	66.07 ± 3.50 ^b^	83.31 ± 5.16 ^a^
Adhesiveness (N·s)	0	2.77 ± 0.52 ^a^	2.90 ± 0.50 ^a^	2.63 ± 0.34 ^a^
	4	0.90 ± 0.17 ^a^	1.23 ± 0.17 ^a^	0.90 ± 0.08 ^a^
	20	0.40 ± 0.08 ^b^	0.83 ± 0.05 ^a^	0.50 ± 0.08 ^b^
	36	0.17 ± 0.05 ^a^	0.20 ± 0.08 ^a^	0.13 ± 0.05 ^a^
Cohesiveness	0	0.40 ± 0.01 ^a^	0.32 ± 0.09 ^a^	0.35 ± 0.02 ^a^
	4	0.56 ± 0.04 ^a^	0.56 ± 0.08 ^a^	0.54 ± 0.02 ^a^
	20	0.61 ± 0.02 ^a^	0.60 ± 0.04 ^ab^	0.53 ± 0.01 ^b^
	36	0.67 ± 0.03 ^a^	0.64 ± 0.04 ^a^	0.69 ± 0.04 ^a^
Springiness	0	0.49 ± 0.02 ^a^	0.48 ± 0.08 ^a^	0.45 ± 0.02 ^a^
	4	0.79 ± 0.02 ^a^	0.83 ± 0.01 ^a^	0.78 ± 0.04 ^a^
	20	0.70 ± 0.02 ^a^	0.71 ± 0.00 ^a^	0.60 ± 0.04 ^a^
	36	0.49 ± 0.04 ^a^	0.50 ± 0.04 ^a^	0.49 ± 0.02 ^a^
Chewiness (N)	0	0.52 ± 0.03 ^a^	0.32 ± 0.09 ^a^	0.46 ± 0.12 ^a^
	4	4.03 ± 0.41 ^a^	4.01 ± 0.87 ^a^	3.97 ± 0.51 ^a^
	20	16.62 ± 0.58 ^a^	16.16 ± 0.44 ^a^	12.88 ± 1.02 ^b^
	36	26.17 ± 2.88 ^ab^	20.93 ± 1.38 ^b^	27.78 ± 1.54 ^a^
Resilience	0	0.12 ± 0.01 ^a^	0.10 ± 0.01 ^a^	0.12 ± 0.01 ^a^
	4	0.24 ± 0.01 ^a^	0.31 ± 0.02 ^a^	0.25 ± 0.02 ^a^
	20	0.23 ± 0.04 ^a^	0.24 ± 0.03 ^a^	0.18 ± 0.04 ^a^
	36	0.24 ± 0.01 ^a^	0.19 ± 0.01 ^b^	0.19 ± 0.01 ^b^
L*	0	53.65 ± 0.47 ^a^	52.97 ± 0.36 ^a^	54.30 ± 1.42 ^a^
	4	58.42 ± 1.22 ^ab^	60.93 ± 1.58 ^a^	57.94 ± 0.33 ^b^
	20	58.55 ± 0.20 ^a^	59.13 ± 0.67 ^a^	54.50 ± 1.09 ^b^
	36	57.27 ± 1.93 ^a^	58.02 ± 0.77 ^a^	53.37 ± 1.08 ^b^
a*	0	7.38 ± 0.73 ^a^	7.47 ± 0.88 ^a^	7.75 ± 0.39 ^a^
	4	10.18 ± 0.30 ^a^	8.01 ± 0.51 ^b^	10.91 ± 1.08 ^a^
	20	10.50 ± 1.47 ^a^	10.37 ± 0.88 ^a^	12.01 ± 0.79 ^a^
	36	12.57 ± 0.55 ^ab^	11.07 ± 1.66 ^b^	14.96 ± 0.84 ^a^
b*	0	10.84 ± 0.86 ^a^	10.21 ± 0.95 ^a^	10.73 ± 0.82 ^a^
	4	9.23 ± 0.72 ^a^	9.15 ± 0.54 ^a^	9.46 ± 0.35 ^a^
	20	7.59 ± 0.58 ^a^	7.21 ± 0.11 ^a^	8.07 ± 0.60 ^a^
	36	6.93 ± 0.78 ^a^	6.42 ± 0.96 ^a^	7.04 ± 0.81 ^a^

N: fermented sausages with 3% NaCl (reference), L: fermented sausages with 2.25% NaCl (25% reduction), R: fermented sausages with partial replacement of 25% NaCl. The average ± standard deviation followed by lowercase letters indicate significant difference (*p* < 0.05) between the samples by Tukey’s test.

**Table 3:** Alpha diversity indexes in samples of the fermented sausages.

Samples	OTU	ACE	Chao1	Simpson	Shannon	Goods Coverage
CK	471.00 ± 87.23 ^a^	580.51 ± 78.15 ^a^	569.34 ± 88.15 ^a^	0.39 ± 0.09 ^b^	2.01 ± 0.46 ^a^	0.996739333
N_D4	132.33 ± 85.20 ^b^	360.33 ± 234.73 ^ab^	246.21 ± 169.80 ^b^	0.75 ± 0.13 ^ab^	0.56 ± 0.15 ^b^	0.998142
N_D20	109.33 ± 52.55 ^b^	334.42 ± 67.32 ^ab^	210.43 ± 48.42 ^b^	0.78 ± 0.29 ^ab^	0.66 ± 0.44 ^b^	0.998409667
N_D36	59.67 ± 15.82 ^b^	182.98 ± 98.70 ^b^	171.61 ± 133.15 ^b^	0.59 ± 0.17 ^ab^	0.82 ± 0.23 ^b^	0.999178333
L_D4	107.67 ± 36.94 ^b^	266.57 ± 54.92 ^ab^	192.21 ± 59.09 ^b^	0.69 ± 0.14 ^ab^	0.86 ± 0.35 ^b^	0.998651
L_D20	74.33 ± 9.29 ^b^	224.67 ± 101.19 ^b^	159.62 ± 49.10 ^b^	0.87 ± 0.04 ^a^	0.37 ± 0.08 ^b^	0.998990333
L_D36	92.67 ± 29.19 ^b^	214.99 ± 112.98 ^b^	155.93 ± 60.69 ^b^	0.87 ± 0.07 ^a^	0.40 ± 0.19 ^b^	0.998973
R_D4	122.00 ± 17.58 ^b^	407.54 ± 202.09 ^ab^	265.98 ± 31.10 ^b^	0.71 ± 0.14 ^ab^	0.59 ± 0.19 ^b^	0.99807
R_D20	71.67 ± 6.66 ^b^	236.96 ± 84.20 ^ab^	128.63 ± 9.21 ^b^	0.71 ± 0.22 ^ab^	0.56 ± 0.29 ^b^	0.998999667
R_D36	70.33 ± 9.29 ^b^	194.60 ± 31.88 ^b^	130.39 ± 12.34 ^b^	0.97 ± 0.00 ^a^	0.12 ± 0.01 ^b^	0.99897266

CK: initial fermented sausages (unfermented), N: fermented sausages with 3% NaCl (reference), L: fermented sausages with 2.25% NaCl (25% reduction), R: fermented sausages with partial replacement of 25% NaCl. The marks D4, D20 and D36 represent the processing times (days). The average ± standard deviation followed by different letters in the same column indicate significant difference (*p* < 0.05) for each sample by Tukey’s test.

## Data Availability

Data is contained within the article or supplementary material.

## Supplementary Materials

The following are available online at https://www.mdpi.com/2304-8158/10/3/630/s1, Figure S1: The response surface three-dimensional diagrams of Box-Behnken experiments of salt replacements; Figure S2: Analysis of the top genera based on the relative abundance in fermented sausage samples; Figure S3: PCoA based on OTUs of the bacterial community among the three groups (N, L and R) of fermented sausages during fermentation and ripening.
