# Supplementary material for: Salt Replacement Changed the Bacterial Community Composition and Physicochemical Characteristics of Sodium-Reduced Fermented Sausages during Fermentation and Ripening
Source: Foods. 2021 Mar 17;10(3):630. doi: 10.3390/foods10030630 (PMC8002409; doi:10.3390/foods10030630)
Supplement: Supplementary file 1 [file foods-10-00630-s001.pdf]

Optimising formula of salt replacements in fermented sausages

Salt replacements formula containing a mixture of KCl, Vc-Ca and calcium glutamate was optimized by using response surface methodology. The response surface three-dimensional diagrams of Box-Behnken experiments of salt replacements were shown in the following Figure S1 below. The salt replacements formula was done using the numerical and graphical optimisation from the software of Design Expert. The optimised mixture was 14 % KCl, 10 % Vc-Ca and 1 % calcium glutamate.

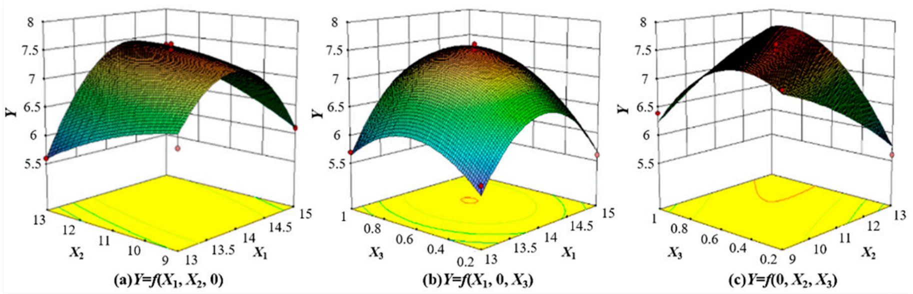

Figure S1. The response surface three-dimensional diagrams of Box-Behnken experiments of salt replacements. X_1、X_2、X_3 were the concentration of KCl, Vc-Ca and glutamate calcium, Y was the sensory acceptability score.

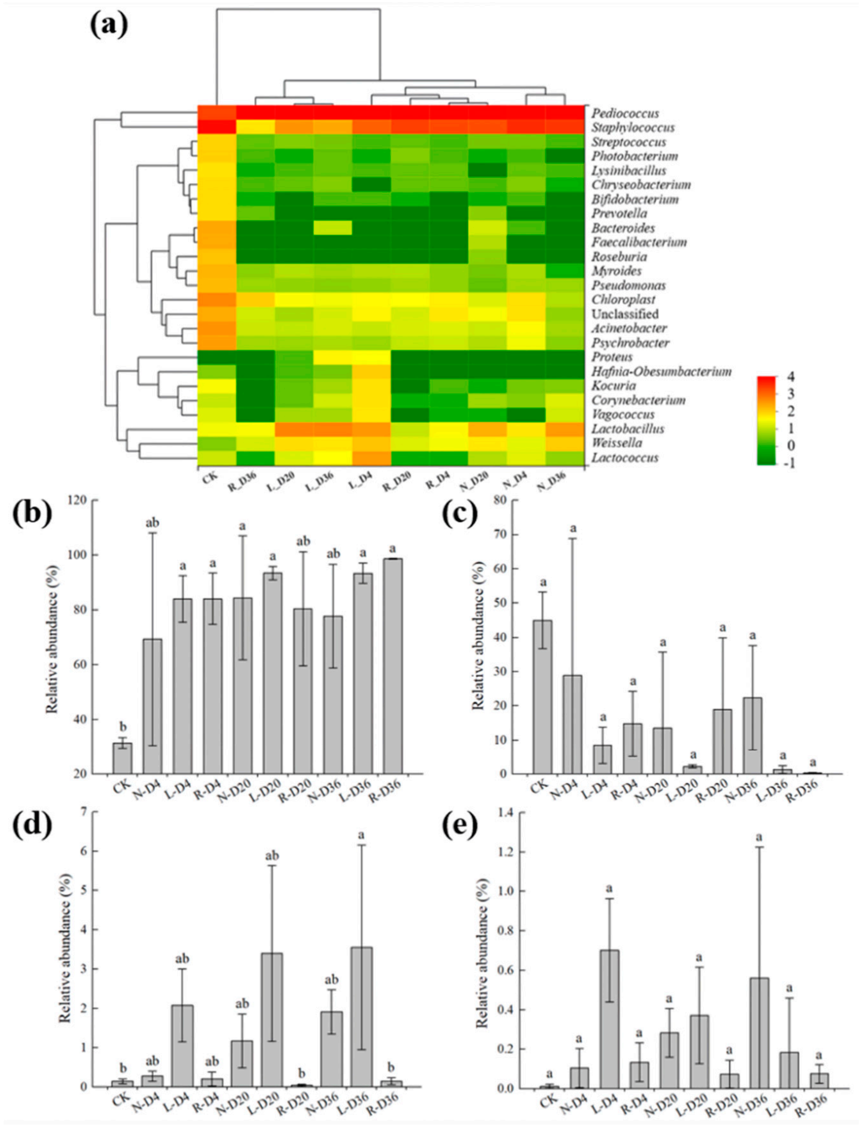

Figure S2. Analysis of the top genera based on relative abundance in fermented sausage samples. (**a**): Heatmap showing the bacterial community structure and composition of the top 25 genera in samples of the fermented sausages. The hierarchical clustering tree is on the left and top based on the genus and sample, respectively. (**b–e**): Relative abundance of *Pediococcus* (**b**), *Staphylococcus* (**c**), *Lactobacillus* (**d**) and *Weissella* (**e**) in fermented sausage samples. CK: initial fermented sausages (unfermented), N: fermented sausages with 3 % NaCl (reference), L: fermented sausages with 2.25 % NaCl (25 % reduction), R: fermented sausages with partial replacement of 25 % NaCl. The marks "D4, D20 and D36" represent the fermentation and ripening times (days). Different letters above the columns indicates a significant difference (p<0.05) for each sample.

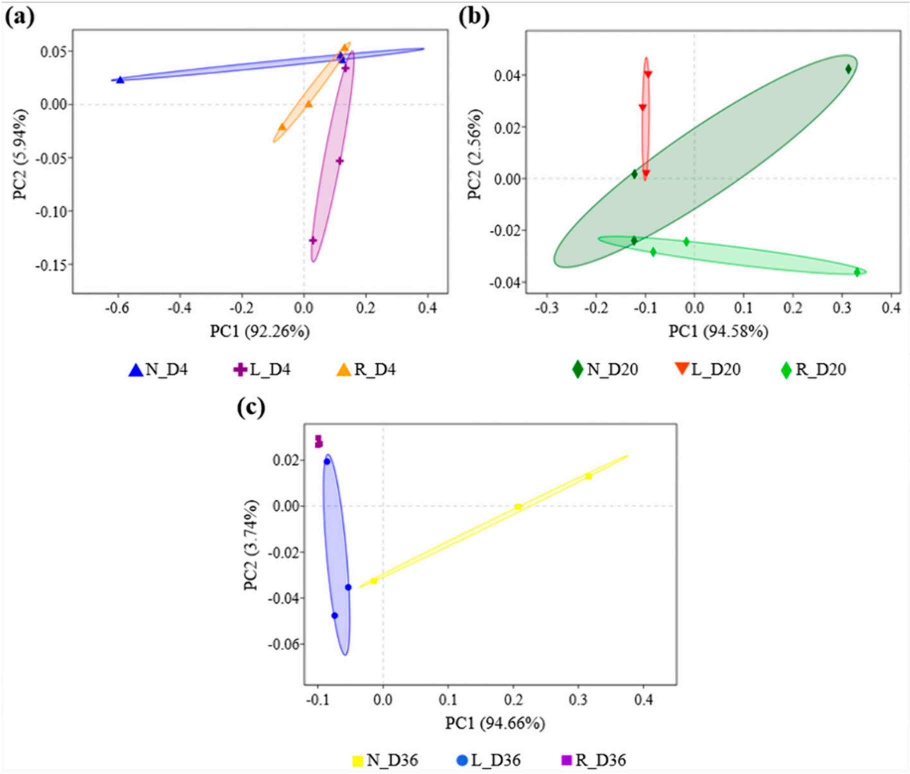

Figure S3. PCoA based on OTUs of the bacterial community among the three groups (N, L and R) of fermented sausages during fermentation and ripening. N: fermented sausages with 3 % NaCl (reference), L: fermented sausages with 2.25 % NaCl (25 % reduction), R: fermented sausages with partial replacement of 25 % NaCl. The marks "D4, D20 and D36" represent the fermentation and ripening times (days).
